# Changes in the Cholinergic, Catecholaminergic, Orexinergic and Serotonergic Structures Forming Part of the Sleep Systems of Adult Mice Exposed to Intrauterine Alcohol

**DOI:** 10.3389/fnana.2017.00110

**Published:** 2017-11-27

**Authors:** Oladiran I. Olateju, Adhil Bhagwandin, Amadi O. Ihunwo, Paul R. Manger

**Affiliations:** School of Anatomical Sciences, Faculty of Health Sciences, University of the Witwatersrand, Johannesburg, South Africa

**Keywords:** fetal alcohol spectrum disorder, brain, sleep, sleep disorders, immunohistochemistry, quantitative studies

## Abstract

We examined the effect of chronic prenatal alcohol exposure on certain neuronal systems involved with the sleep-wake cycle of C57BL/6J mice exposed to prenatal alcohol once they had reached 56 days post-natal. Pregnant mice were exposed to alcohol, through oral gavage, on gestational days 7–16, with recorded blood alcohol concentration (BAC)s averaging 1.84 mg/ml (chronic alcohol group, CA). Two control groups, an oral gavage sucrose control group (chronic alcohol control group, CAc) and a non-treated control group (NTc), were also examined. At 56 days post-natal, the pups from each group were sacrificed and the whole brain sectioned in a coronal plane and immunolabeled for cholineacetyltransferase (ChAT), tyrosine hydroxylase (TH), serotonin (5HT) and orexin-A (OxA) which labels cholinergic, catecholaminergic, serotonergic and orexinergic structures respectively. The overall nuclear organization and neuronal morphology were identical in all three groups studied, and resemble that previously reported for laboratory rodents. Quantification of the estimated numbers of ChAT immunopositive (+) neurons of the pons, the TH+ neurons of the pons and the OxA+ neurons of the hypothalamus showed no statistically significant difference between the three experimental groups. The stereologically estimated areas and volumes of OxA+ neurons in the CA group were statistically significantly larger than the groups not exposed to prenatal alcohol, but the ChAT+ neurons in the CA group were statistically significantly smaller. The density of orexinergic boutons in the anterior cingulate cortex was lower in the CA group than the other groups. No statistically significant difference was found in the area and volume of TH+ neurons between the three experimental groups. These differences are discussed in relation to the sleep disorders recorded in children with fetal alcohol spectrum disorder (FASD).

## Introduction

Adequate quality sleep, especially in children, is important for the development of the brain (Chen et al., 2012; Ipsiroglu et al., 2013). Certain neural functions such as learning, cognition, motor activities, attention, alertness, as well as brain growth and metabolic activities, are only performed effectively if there has been adequate sleep (Chen et al., 2012; Ipsiroglu et al., 2013). Sleep problems are often observed in concert with neurodevelopmental conditions such as autism (Malow et al., 2006), attention deficit hyperactivity disorders (Cortese et al., 2009) and fetal alcohol syndrome (FAS; Streissguth et al., 2004; May et al., 2009; Olson et al., 2009; Chen et al., 2012; Ipsiroglu et al., 2013). Children with FAS show an unwillingness to go to bed (Meltzer and Mindell, 2004; Wengel et al., 2011), experience short sleep duration (Jan et al., 2010; Wengel et al., 2011), and experience sleep anxiety with frequent sleep disruptions (Haydon et al., 2009; Wengel et al., 2011). Moreover, FAS children report night terrors (Durmer and Dinges, 2005), exhibit sleep walking (Randazzo et al., 1998), daytime tiredness (Lancioni et al., 1999) and suppressed sensory information processing (Wengel et al., 2011) when compared to neurotypical children. Similarly, in laboratory rodents exposed to intrauterine alcohol, polysomnographic recording of the sleep-wake cycle demonstrated that total sleep duration was significantly reduced, with a concomitant increase in total wake time, and increases in the numbers of both sleep and wake episodes (Hilakivi, 1986; Stone et al., 1996).

The neural systems that control and regulate sleep are comprised of neurons in specific nuclear clusters in the basal forebrain, hypothalamus and pons, which produce a variety of neurotransmitters and project throughout the brain. These neurons depolarize in specific patterns during wake, slow wave sleep (SWS) and rapid eye movement sleep (REM; Datta and MacLean, 2007; Lyamin et al., 2008; Takahashi et al., 2010; Dell et al., 2012; Bhagwandin et al., 2013; Petrovic et al., 2013). Due to the oscillatory relationship of these neuronal groups in the sleep-wake cycle, it may be that the anatomy of these neuronal systems differ from neurotypical in FAS children with sleep disorders; however, to the author’s knowledge, there appear to be no reports that directly correlate morphological changes in the sleep centers in FAS subjects that are known to experience sleep disorders. In order to determine whether morphological changes in the neural systems involved in the production and regulation of sleep may be correlated to the sleep disorders observed in FAS children, we targeted for investigation the organization, morphology and numbers of pontine cholinergic (laterodorsal tegmental, LDT and pedunculopontine nuclei), pontine catecholaminergic (locus coeruelus complex, LC), hypothalamic orexinergic and midbrain serotonergic (dorsal raphe) neurons of 56 day old C57BL/6J mice following exposure to prenatal alcohol. In addition to qualitative examination of these neurons involved in sleep, stereological analysis of neuronal numbers and neuronal size was undertaken for the cholinergic LDT and pedunculopontine tegmental nucleus (PPT), the noradrenergic LC, the orexinergic neurons of the hypothalamus, and orexinergic bouton density in the anterior cingulate cortex.

## Materials and Methods

### Breeding and Prenatal Ethanol Exposure

All animals were treated and used according to the guidelines of the University of the Witwatersrand Animal Ethics Committee (Clearance No. 2012/15/2B), which parallel those of the NIH for the care and use of animals in scientific experimentation. Female C57BL/6J mice (*Mus musculus*), 12 weeks of age, were allocated into three experimental groups: Chronic Alcohol exposure (CA), control for Chronic Alcohol exposure (CAc) and a Non-Treatment control group (NTc). For effective mating, 1–2 female mice were introduced into the cage of a C57BL/6J male mouse for 12 h, which was considered gestational day 0 (GD 0). In all, a total of 14 female mice (4–5 mice assigned to each experimental group) and 8 male mice were used to generate the required numbers of pups used in the present study.

For the CA group, each pregnant mouse received a dose of 7.5 μl/g of 50% alcohol in distilled water (2.9 g/kg) per day (Haycock and Ramsay, 2009; Knezovich and Ramsay, 2012) for 10 consecutive days by oral gavage, starting from GD 7 (Webster et al., 1980, 1983; Sulik et al., 1981; Choi et al., 2005; Redila et al., 2006; Parnell et al., 2009), while each pregnant mouse in the CAc group received an equivalent dose of isocaloric sucrose (704 g/L) by oral gavage over the same period (Haycock and Ramsay, 2009; Knezovich and Ramsay, 2012). To control for the possible influence of post-traumatic stress in the pregnant mice, pregnant mice in the NTc group did not undergo any oral gavage. Food and water was provided *ad libitum* to the mice, except in the control groups (CAc and NTc), where it was withheld for 2 h post-gavage in order to partially control for the reduction in feeding during the period of peak intoxication of the alcohol-treated dams (Haycock and Ramsay, 2009). The pups were weaned 21 days after birth and then the male and female pups separated. Three pups of the same sex from each experimental group were kept in separate cages (cage dimensions: 200 × 200 × 300 mm) with adequate food and water supplies until post-natal day (PND) 56.

### Blood Alcohol Concentration Assay in the Pregnant Mice

On the last day (10th day) of oral gavage (GD 16), a small lesion was made at the site of the saphenous vein on the left hind legs of all the pregnant mice in the CA and CAc experimental groups. The saphenous bleeding procedure was performed on the pregnant mice in the sucrose group in order to mimic the effects of the bleeding on the alcohol exposed pregnant mice. The non-treatment pregnant mice served as controls for the possible effects of the bleeding and/or the oral gavage procedures. Fifty microliter of blood was drawn into heparinized capillary tubes at 30 min post-gavage (Bielawski and Abel, 1997) to determine the blood alcohol concentration (BAC). The blood samples from the FAS model and the sucrose control were stored at 4°C overnight after which they were centrifuged with Vivaspin500 100 μm membrane tubes (Biotech, South Africa) for 30 min before the serum was extracted and the BAC analyzed using an EnzyChrom™ Ethanol Assay Kit (BioVision, South Africa). The pregnant mice belonging to the CA group that were administered alcohol had an average BAC of 1.84 mg/ml (s.e. = 0.39), which is above the pharmacologically significant level of 1 mg/ml reported by Rhodes et al. (2005) and Sulik (2005).

### Sacrifice and Tissue Processing

At PND 56, when the mice reached adulthood, a total number six mice (*n* = 6) from each experimental group (1–2 mice randomly selected from each litter to control for potential genetic influences) were weighed and then euthanized (Eutha-naze 1 ml/kg, contains sodium pentobarbitone 100 mg/ml, intra-peritoneally) before being perfused trans-cardially with 0.9% cold (4°C) saline followed immediately by cold 4% paraformaldehyde in 0.1 M phosphate buffer (PB). The brain was removed from the skull, weighed and post-fixed for 24 h in 4% paraformaldehyde in 0.1 M PB at 4°C. The brains were then cryoprotected by immersion in 30% buffered sucrose solution in 0.1 M PB at 4°C until they equilibrated. The brains for all 18 mice was then frozen in crushed dry ice, and sectioned in a coronal plane at 50 μm thickness using a sliding microtome. A one in four series of sections was taken. The first series of sections, stained for Nissl substance to reveal cytoarchitectural features, was mounted on 0.5% gelatine-coated slides, dried overnight, cleared overnight in a 1:1 mixture of 100% ethanol and 100% chloroform and stained with 1% cresyl violet in H_2_O. The second series of sections was immunostained with an antibody to cholineacetyltransferase (ChAT, AB144P, Chemicon, raised in goat) to reveal cholinergic neurons, and the third series was immunostained with an antibody to tyrosine hydroxylase (TH; AB151, Chemicon, raised in rabbit) to reveal catecholaminergic neurons. The fourth series of sections was divided at the level of the posterior commissure. All sections rostral to the posterior commissure were immunostained with an antibody to orexin-A (OxA, AB3704, Merck-Millipore, raised in rabbit) to reveal the hypothalamic orexinergic neurons and orexinergic boutons in the cerebral cortex, while all section caudal to the posterior commissure were immunostained with an antibody to serotonin (5HT, AB938, Chemicon, raised in rabbit) to reveal the serotonergic neurons.

### Immunostaining Protocol

All sections used for immunohistochemical staining were initially incubated in a solution containing 1.6% of 30% H_2_O_2_, 49.2% methanol and 49.2% 0.1 M PB solution, for 30 min to reduce endogenous peroxidase activity, which was followed by three 10 min rinses in 0.1 M PB. To block non-specific binding sites the sections were then preincubated at room temperature for 2 h in a blocking buffer solution containing 3% normal serum (normal rabbit serum, NRS, Chemicon, for ChAT sections and normal goat serum, NGS, Chemicon, for the remaining sections), 2% bovine serum albumin (BSA, Sigma) and 0.25% Triton X-100 (Merck) in 0.1 M PB. The sections were then placed in a primary antibody solution (blocking buffer with correctly diluted primary antibody) and incubated at 4°C for 48 h under gentle shaking. To reveal cholinergic neurons, anti-ChAT at a dilution of 1:3000 was used. To reveal putative catecholaminergic neurons, anti-TH was used at a dilution of 1:3000. To reveal serotonergic neurons, anti-5-HT at a dilution of 1:5000 was used. To reveal hypothalamic orexinergic neurons and cortical orexinergic boutons, anti-OxA at a dilution of 1:3000 was used.

This was followed by three 10 min rinses in 0.1 M PB, after which the sections were incubated in a secondary antibody solution for 2 h at room temperature. The secondary antibody solution contained a 1:1000 dilution of biotinylated anti-goat IgG (BA-5000, Vector labs, for ChAT sections) or biotinylated anti-rabbit IgG (BA-1000, Vector Labs, for the remaining sections) in a solution containing 3% NGS/NRS and 2% BSA in 0.1 M PB. This was followed by three 10 min rinses in 0.1 M PB after which the sections were incubated in an avidin-biotin solution (Vector Labs) for 1 h. After three further 10 min rinses in 0.1 M PB, the sections were placed in a solution of 0.05% diaminobenzidine in 0.1 M PB for 5 min (1 ml/section), followed by the addition of 3 μl of 30% H_2_O_2_ to the solution in which each section was immersed. The precipitation process was stopped by immersing the sections in 0.1 M PB and then rinsing them twice more in 0.1 M PB. To check for non-specific staining from the immunohistochemistry protocol, we omitted the primary antibody and the secondary antibody in selected sections, which produced no evident staining. The sections were then mounted on 0.5% gelatine coated glass slides, dried overnight, dehydrated in a graded series of alcohols, cleared in xylene and coverslipped with DPX.

### Qualitative and Quantitative Determination of Cell Numbers and Statistical Analysis

The distribution of immunopositive cells was compared qualitatively between the experimental groups using both low and higher power light microscopy. Digital photomicrographs of these cells were captured using Zeiss Axioshop and Axiovision software. No pixelation adjustments or manipulation of the captured images was undertaken, except for the adjustment of contrast, brightness and levels using Adobe Photoshop 7.

For the quantification of Chat+, TH+, OxA+ cells and OxA+ boutons, an unbiased systematic random sampling stereological design protocol was employed. We used a MicroBrightfield (MBF; Colchester, VT, USA) system with three plane motorized stage, Zeiss.Z2 vario axioimager and StereoInvestigator software (MBF, version 11.08.1; 64-bit). Separate pilot studies for each immunohistochemical stain for each group, were conducted to optimize sampling parameters, such as the counting frame and sampling grid sizes, and achieve a coefficient of error (CE) below 0.1 (Gundersen et al., 1988; West et al., 1991; Dell et al., 2016). At this point we would like to acknowledge that while every effort was made to obtain a CE below 0.1, this was not always achieved mostly due to a limited number of countable sections in those instances. In addition we measured the tissue section thickness at every 10th sampling site and the vertical guard zone was determined according to tissue thickness to avoid errors/biases due to sectioning artifacts (West et al., 1991; Dell et al., 2016). We decided to maintain consistency amongst sampling parameters between the groups studied for each neuroanatomical region and employed a single-blind procedure, to reduce unfavorable stereological estimation biases. Table 1 provides a detailed summary of the parameters used for each neuroanatomical region and between the groups in the current study. Finally, with regard to the OxA+ bouton densities, a region of interest (ROI) measuring 1200 × 800 μm encompassing all six layers in similar locations of the anterior cingulate cortex in all three groups was used to calculate the densities reported.

**Table 1:** Stereological parameters used for estimating cell numbers, cell densities and cell sizes in the various nuclei quantified in the current study.

Experimental group	Antibody	Counting frame size (μm)	Grid frame size (μm)	Disector height (μm)	Section thickness (μm)	Average mounted thickness (μm)	Guard zones (μm)	Section interval	Average number of sections	Average number of sampling sites	Average CE (Gundersen *m* = 1)
**Cholinergic neurons of the laterodorsal tegmental (LDT) and pedunculopontine tegmental (PPT) nuclei**											
CA	ChAT	300 × 300	300 × 300	11	50	17.6	2	4	10.2	159.4	0.07
CAc	ChAT	300 × 300	300 × 300	11	50	16.8	2	4	11.3	182.2	0.08
NTc	ChAT	300 × 300	300 × 300	11	50	16.7	2	4	12.8	180.6	0.07
**Noradrenergic neurons of the locus coeruleus complex**											
CA	TH	375 × 375	375 × 375	11	50	16.3	2	4	5.2	60.8	0.14
CAc	TH	375 × 375	375 × 375	11	50	17.5	2	4	4.8	68.2	0.15
NTc	TH	375 × 375	375 × 375	11	50	17.6	2	4	5.5	66.3	0.17
**Orexinergic neurons of the hypothalamus**											
CA	OxA	375 × 375	375 × 375	13	50	18.2	2	4	4.4	60.6	0.13
CAc	OxA	375 × 375	375 × 375	13	50	20.9	2	4	4.2	51.3	0.14
NTc	OxA	375 × 375	375 × 375	13	50	18.4	2	4	4.0	102.0	0.14
**Orexinergic boutons in the cerebral cortex**											
CA	OxA	400 × 400	233.3 × 233.3	16	50	20.7	2	2	6	63.8	0.03
CAc	OxA	400 × 400	233.3 × 233.3	18	50	22.8	2	2	6	62.0	0.03
NTc	OxA	400 × 400	233.3 × 233.3	17	50	22.1	2	2	6	62.0	0.03

*CA, chronic alcohol group; CAc, control for chronic alcohol group; NTc, non-treated control group; CE, coefficient of error*.

To estimate the total number of pontine Chat+ neurons (LDT and PPT), pontine locus coeruleus TH+ neurons, hypothalamic OxA+ neurons and cortical OxA+ boutons, we used the “Optical Fractionator” probe and the following equation (West et al., 1991; Dell et al., 2016):

(1) N=Q/(SSF×ASF×TSF)

where *N* was the total estimated neuronal number, *Q* was the number of neurons counted, *SSF* was the fraction of the sections sampled, *ASF* was the area sub fraction (which is calculated by the ratio of the size of the counting frame to the size of the sampling grid), and *TSF* was the thickness sub fraction (which is calculated by the ratio of the disector height relative to the section thickness).

To determine neuronal sizes, we used the “Nucleator” probe. For all individuals counted these probes were used concurrently while maintaining strict criteria, e.g., only neurons with complete cell bodies were counted, and obeying all commonly known stereological rules.

An analysis of variance (ANOVA) was performed to determine if there was a significant variation in the mean ChAT+, TH+, or OxA+ neuronal counts, areas and volumes of all the experimental groups (CA, CAc and NTc). In addition, ANOVA was used to determine sex differences in the mean ChAT+, TH+, or OxA+ neuronal counts, areas and volumes within and/or between the sexes within and between groups. Where the ANOVA was significant, a *post hoc* analysis using Tukey’s pairwise comparison revealed where significant differences existed. In this report, only statistically significant differences in the mean neuronal counts, areas and volumes are reported in the results. All statistical analyses were performed using SPSS Inc programme (version 22.0). A significance level of 5% was used as an indicator of significant differences for all statistical analyses.

## Results

### General Observations on the Body and Brain

The pups that experienced chronic prenatal alcohol exposure (CA group) showed no overt signs of FAS, in that no craniofacial abnormalities were readily apparent and there was no evident reduction in overall body mass. At the time of sacrifice, the average body masses of the mice were: CA male—19.75 g (s.e. 0.75 g), CA female—15.13 g (s.e. 0.55 g); CAc male— 19.88 g (s.e. 0.4 g), CAc female—16.00 g (s.e. 0.29 g); NTc male—20.25 g (s.e. 0.75 g), NTc female— 15.63 g (s.e. 0.24 g). In addition, there were no observable differences in the general morphology of the brains of mice treated with alcohol (CA group), sucrose (CAc group) or the non-treated control group (NTc). The average brain masses recorded for each group were: CA male—0.420 g (s.e. 0.02 g), CA female—0.390 g (s.e. 0.007 g); CAc male—0.396 g (s.e. 0.005 g), CAc female—0.376 g (s.e. 0.003 g); NTc male—0.400 g (s.e. 0.006 g), NTc female—0.390 g (s.e. 0.007 g). No statistically significant differences were observed between experimental groups in terms of body or brain mass when mice of the same sex were compared.

### Cholinergic Neurons of the Laterodorsal Tegmental and Pedunculopontine Nuclei

**Figure 1:**
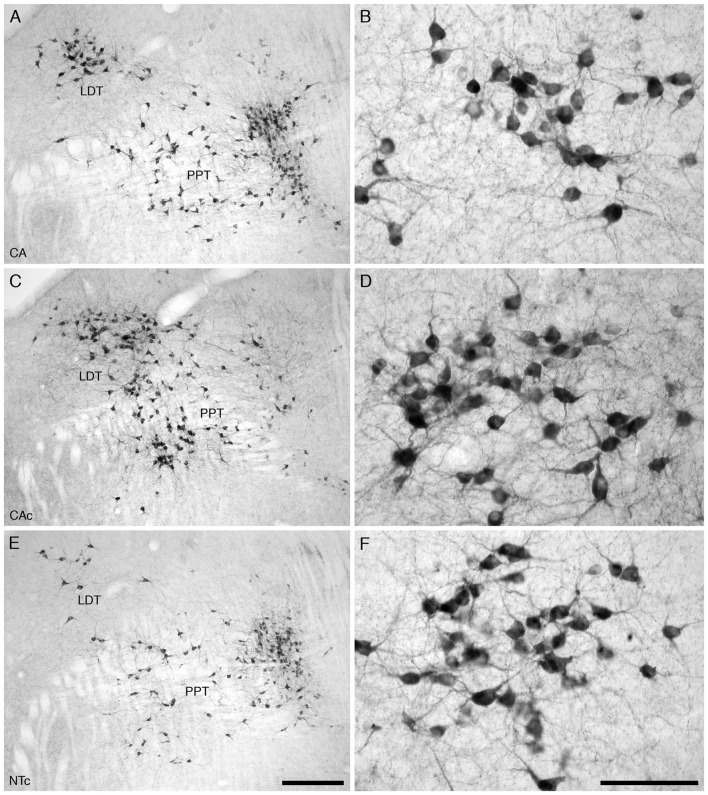
Lower **(A,C,E)** and higher **(B,D,F)** magnification photomicrographs of the laterodorsal tegmental (LDT; **B,D,F**) and pedunculopontine (PPT) nuclei of the mouse in the coronal plane immunostained for cholineacetyltransferase (ChAT) in the three different groups analyzed in the present study, the group exposed to chronic prenatal alcohol (CA; **A,B**), the prenatal gavage control group (CAc; **C,D**) and the non-treated control group (NTc; **E,F**). The nuclear organization and number of ChAT immunostained cells was similar between groups, but the soma of the neurons in the CA group, exposed to chronic prenatal alcohol, were statistically significantly smaller than the two control groups (CAc and NTc). In all images dorsal is to the top and medial to the left. Scale bar in **(E)** = 250 μm and applies to **(A,C,E)** scale bar in **(F)** = 100 μm and applies to **(B,D,F)**.

For all mice in all three experimental groups, there were no marked differences in the location and morphology of the ChAT+ neurons within the PPT and the LDT nuclei. The ChAT+ neurons forming the PPT were located within the dorsal aspect of the pontine tegmentum extending from the ventrolateral border of the periaqueductal/periventricular gray matter to the superior cerebellar peduncle (Figure 1). A moderate to high density of ChAT+ neurons were observed in this region. The PPT and LDT ChAT+ neurons exhibited a variety of somal shapes due to being multipolar (Figure 1).

**Figure 2:**
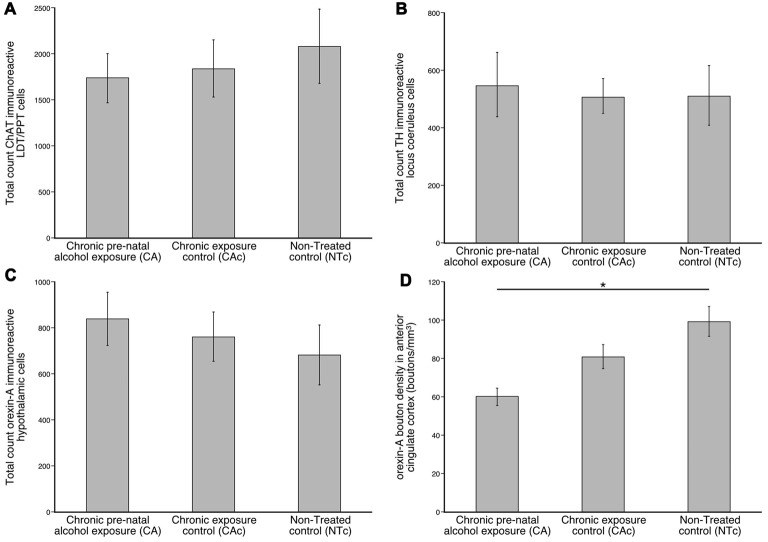
Graphs showing the average numbers of cholinergic neurons (ChAT; **A**), catecholaminergic neurons (TH; **B**), orexinergic neurons **(C)**, and density of orexinergic boutons in the anterior cingulate cortex **(D)** in the brains of three different groups (*n* = 6 per group) analyzed in the present study, the group exposed to chronic prenatal alcohol (CA), the prenatal gavage control group (CAc) and the NTc. **(A)** Note that while the numbers of ChAT immunoreactive neurons in the LDT and PPT nuclei are lower in the CA group, this difference is not statistically significant. **(B)** Note that while the numbers of TH immunoreactive neurons in the locus coeruleus complex (LC) is higher in the CA group, this difference is not statistically significant. **(C)** Note that while the numbers of orexin-A (OxA) immunoreactive neurons in the hypothalamus is higher in the CA group, this difference is not statistically significant. **(D)** Note that the density of orexinergic boutons in the anterior cingulate cortex is lower in the CA group than the other groups, and this is statistically significantly lower than the untreated control (NTc) group *p = 0.002.

Our quantitative estimation of the numbers of ChAT+ neurons in the LDT and PPT in the brain of mice from the three different experimental groups revealed a distinct homogeneity in numbers between individuals in the same group, and between groups. For the CA group, the average number of ChAT+ neurons was 1741.4 (s.e. 276.8) (male: 1933.2 (s.e. 427.8), female: 1549.6 (s.e. 450.8)), for the CAc group it was 1844.3 (s.e. 312.7) (male: 1686.9 (s.e. 593.5), female: 1646.1 (s.e. 311.9)) and for the NTc group it was 2091.9 (s.e. 405.2) (male: 1256.0 (s.e. 355.0), female: 2961.4 (s.e. 62.6); Figure 2A). While there is a trend for the CA and CAc groups to have less ChAT+ neurons than the NTc group, these were not statistically significant differences. ANOVA and *post hoc* pairwise comparisons revealed that the lower number of ChAT+ neurons in the CA group was not statistically significantly different from the CAc group (*p* = 0.977) or the NTc group (*p* = 0.763). A comparison between CAc and NTc groups was also not statistically significantly different (*p* = 0.872).

**Figure 3:**
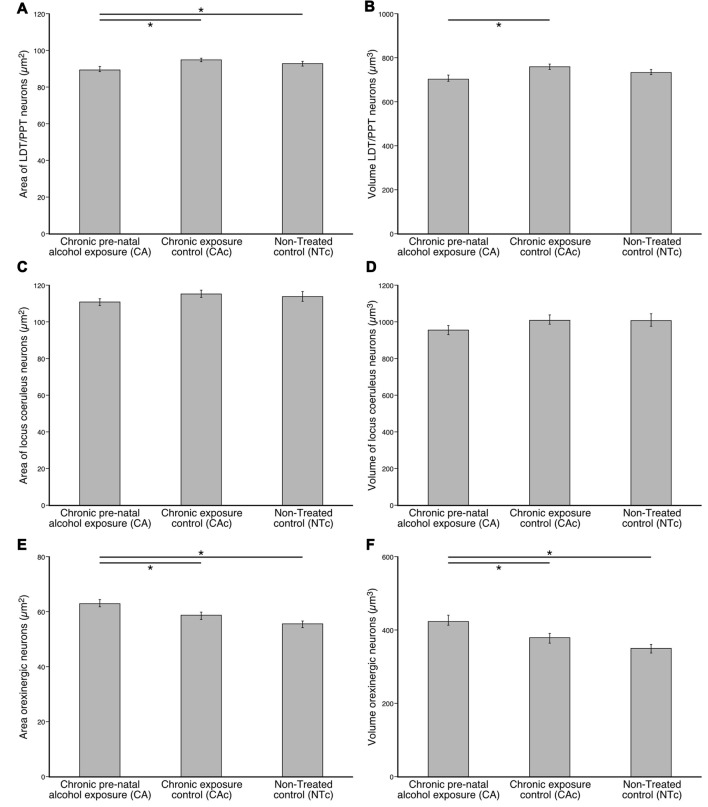
Graphs showing the average somal area **(A,C,E)** and average somal volume **(B,D,F)** of cholinergic (ChAT; **A,B**), catecholaminergic (TH; **C,D**) and orexinergic (OxA; **E,F**) immunoreactive cells in the brains of three different groups (*n* = 6 per group) analyzed in the present study, the group exposed to chronic prenatal alcohol (CA), the prenatal gavage control group (CAc) and the NTc. The average somal area and volume of the ChAT+ neurons of the LDT and PPT nuclei **(A,B)** in the CA group are statistically significantly (*) smaller than the control groups (CAc: *p*_area_ = 4 × 10^−5^; *p*_volume_ = 4 × 10^−5^ respectively and NTc: *p*_area_ = 0.017; *p*_volume_ = 0.070). The average somal area and volume of the TH+ neurons of the LC **(C,D)** are not statistically significantly different between the three groups. The average somal area and volume of the OxA+ neurons of the hypothalamus **(E,F)** in the CA group are statistically significantly (*) larger than the control groups (CAc: *p*_area_ = 0.026; *p*_volume_ = 0.030) and NTc: *p*_area_ = 5 × 10^−5^; *p*_volume_ = 9 × 10^−5^).

In terms of average somal area, the CA group had an average somal area of 89.7 μm^2^ (s.e. 1.0) (male: 78.8 (s.e. 1.3), female: 100.5 (s.e. 1.6)), for the CAc group it was 95.0 μm^2^ (s.e. 0.7) (male: 100.1 (s.e. 0.9), female: 86.4 (s.e. 1.1)) and for the NTc group it was 92.9 μm^2^ (s.e. 0.8) (male: 98.9 (s.e. 1.4), female: 90.0 (s.e. 0.9); Figure 3A). Statistical analyses using ANOVA and *post hoc* pairwise comparisons revealed that the smaller somal area of the ChAT+ neurons in the CA group was statistically significantly smaller than the CAc group (*p* = 3.8 × 10^−5^) and the NTc group (*p* = 0.017). A comparison of somal areas between the CAc and NTc groups was not statistically significantly different (*p* = 0.170). In regards to average somal volume, the CA group exhibited an average somal volume of 707.5 μm^3^ (s.e. 12.5) (male: 572.1 (s.e. 14.9), female: 827.9 (s.e. 20.0)), for the CAc group it was 763.9 μm^3^ (s.e. 8.9) (male: 823.0 (s.e. 16.1), female: 663.9 (s.e. 13.1)) and for the NTc group it was 739.4 μm^3^ (s.e. 9.4) (male: 816.4 (s.e. 18.2), female: 703.3 (s.e. 10.8); Figure 3B). Statistical analyses revealed that the smaller somal volumes of the ChAT+ neurons in the CA group was statistically significantly smaller than the somal volumes of the CAc group (*p* = 3 × 10^−4^), but not significantly different from the NTc group (*p* = 0.070). A comparison of the somal volumes between the CAc and NTc groups was not statistically significantly different (*p* = 0.208). Thus, for the pontine cholinergic neurons, the only substantive difference observed between the groups was the reduction in size, by around 5%–10%, of the soma in the group exposed to prenatal alcohol.

When comparing the two sexes, the somal areas and volumes in the CA group was statistically significantly different from the CAc (*p* = 2.2 × 10^−5^) and NTc (*p* = 2.2 × 10^−5^) groups. There was similarly sex differences between the two sexes in each experimental group for the somal areas (CA_male_ vs. CA_female_ : *p* = 2 × 10^−5^; CAc_male_ vs. CAc_female_ : *p* = 2 × 10^−5^; NTc_male_ vs. NTc_female_ : *p* = 3.5 × 10^−5^) and somal volumes (CA_male_ vs. CA_female_ : *p* = 2 × 10^−5^; CAc_male_ vs. CAc_female_ : *p* = 2 × 10^−5^; NTc_male_ vs. NTc_female_ : *p* = 2.6 × 10^−5^).

### Catecholaminergic Neurons of the Locus Coeruleus Complex

**Figure 4:**
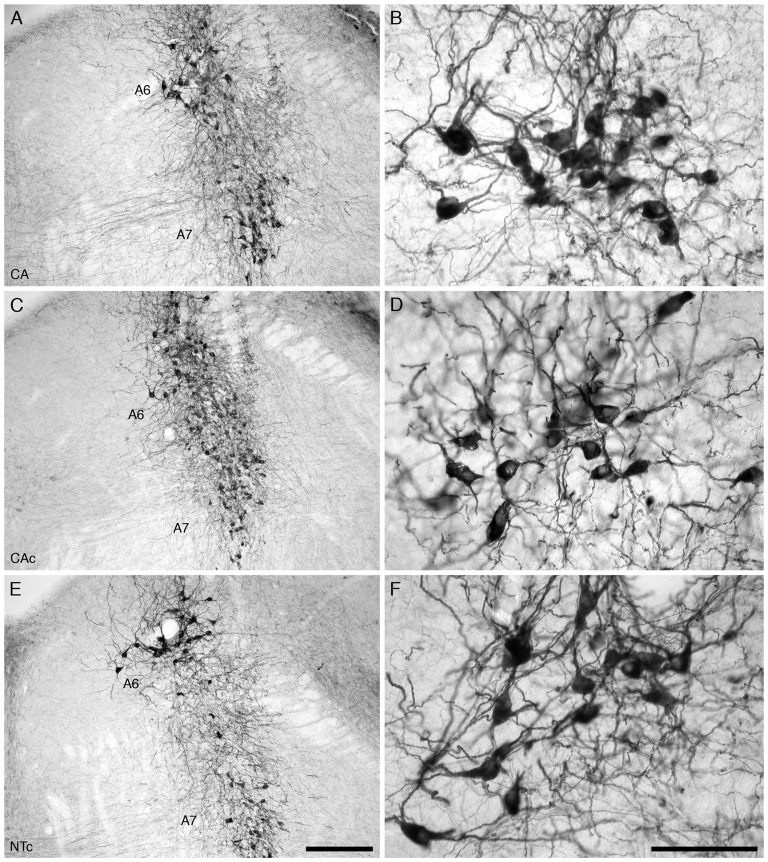
Lower **(A,C,E)** and higher **(B,D,F)** magnification photomicrographs of the locus coeruleus (A6, **B,D,F**) and subcoeruleus (A7) nuclei of the mouse in the coronal plane immunostained for tyrosine hydroxylase (TH) in the three different groups analyzed in the present study, the group exposed to chronic prenatal alcohol (CA; **A,B**), the prenatal gavage control group (CAc; **C,D**) and the NTc **(E,F)**. The nuclear organization, neuronal morphology, somal size and number of TH immunostained cells in the LC was similar between groups. In all images dorsal is to the top and medial to the left. Scale bar in **(E)** = 250 μm and applies to **(A,C,E)** scale bar in **(F)** = 100 μm and applies to **(B,D,F)**.

For all mice in all three experimental groups, there were no marked differences in the location and morphology of the TH+ neurons within the LC of the pontine region, where the TH+ neurons forming the locus coeruleus were readily observed (Figure 4). This complex contained five nuclei: the subcoeruleus compact portion (A7sc), subcoeruleus diffuse portion (A7d), locus coeruleus compact portion (A6c), fifth arcuate nucleus (A5) and the dorsolateral division of locus coeruleus (A4). The distribution of the neurons forming these five nuclear subdivisions of the LC was the same as what has been previously described in other laboratory rodents (Dahlström and Fuxe, 1964; Olson and Fuxe, 1972), thus an extensive description is not provided here. All neurons throughout the LC showed a similar variety of somal shapes and all were multipolar.

Our quantitative estimation of the numbers of TH+ neurons in the A6 and A7 nuclei of the LC from the three different experimental groups revealed a distinct homogeneity in numbers between individuals in the same group, and between groups (Figure 2B). For the CA group, the average number of TH+ neurons was 549.9 (s.e. 113.6) (male: 424.8 (s.e. 180.5, female: 674.9 (s.e. 116.4)), for the CAc group it was 510.2 (s.e. 60.6) (male: 576.9 (s.e. 38.0), female: 465.7 (s.e. 96.4)) and for the NTc group it was 512.8 (s.e. 104.4) (male: 409.2 (s.e. 148.5), female: 668.2 (s.e. 51.8); Figure 2B). While the number of TH+ neurons is highest in the CA group, statistical analyses using ANOVA and *post hoc* pairwise comparisons revealed that the number of TH+ neurons in the CA group was not statistically significantly different from the CAc group (*p* = 0.952) or the NTc group (*p* = 0.958). A comparison between CAc and NTc groups was also not statistically significantly different (*p* = 0.999).

In terms of average somal area, the CA group had an average somal area of 111.1 μm^2^ (s.e. 1.9) (male: 109.8 (s.e. 2.2), female: 113.8 (s.e. 3.6)), for the CAc group it was 115.5 μm^2^ (s.e. 1.9) (male: 113.9 (s.e. 2.4), female: 118.2 (s.e. 3.0)) and for the NTc group it was 114.1 μm^2^ (s.e. 2.5) (male: 139.1 (s.e. 5.1), female: 101.7 (s.e. 2.3); Figure 3C). Statistical analyses using ANOVA and *post hoc* pairwise comparisons revealed that the average somal areas of the TH+ neurons in the CA group was not statistically significantly different from the CAc group (*p* = 0.305) or the NTc group (*p* = 0.578). A comparison between CAc and NTc groups was also not statistically significantly different (*p* = 0.884). In regards to average somal volume, the CA group exhibited an average somal volume of 955.5 μm^3^ (s.e. 25.2) (male: 937.5 (s.e. 29.5), female: 991.9 (s.e. 47.1)), for the CAc group it was 1011.3 μm^3^ (s.e. 25.6) (male: 993.8 (s.e. 32.7), female: 1041.3 (s.e. 40.8)) and for the NTc group it was 1007.1 μm^3^ (s.e. 33.7) (male: 1347.6 (s.e. 72.4), female: 838.7 (s.e. 28.7); Figure 3D). Statistical analyses revealed that the average somal volume of the TH+ neurons in the CA group was not statistically significantly different from the CAc group (*p* = 0.340) or the NTc group (*p* = 0.397). A comparison between CAc and NTc groups was similarly not statistically significantly different (*p* = 0.994). Thus, for the neurons of the LC, no differences in nuclear organization, neuronal morphology, neuronal numbers, or somal size was detected between the three experimental groups investigated.

When analyzing the two sexes independently, the somal areas for the NTc group were significantly different from the CA group (male: *p* = 2 × 10^−5^; female: *p* = 0.010) and the CAc group (male: *p* = 2 × 10^−5^; female: *p* = 2 × 10^−4^). Likewise, somal volume in the NTc group was significantly different from the CA group (male: *p* = 2 × 10^−5^; female: *p* = 0.010) and the CAc group (male: *p* = 2 × 10^−5^; female: *p* = 5 × 10^−4^). When comparing between the sexes within the same group, the somal areas and volumes in the NTc group (i.e., NTc_male_ vs. NTc_female_) were significantly different (somal area: *p* = 2 × 10^−5^; somal volume: *p* = 2 × 10^−5^).

### Serotonergic Neurons of the Dorsal Raphe Nuclear Complex

**Figure 5:**
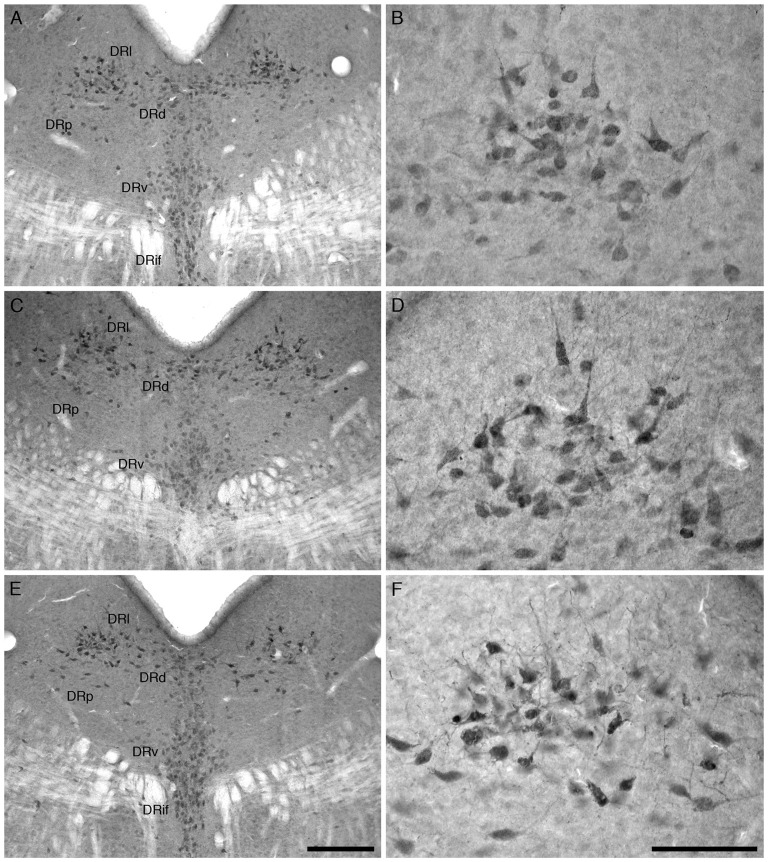
Lower (**A,C,E**, several nuclei) and higher (**B,D,F**, DRl) magnification photomicrographs of the dorsal raphe nuclear complex (DR) of the mouse in the coronal plane immunostained for serotonin (5-HT) in the three different groups analyzed in the present study, the group exposed to chronic prenatal alcohol (CA; **A,B**), the prenatal gavage control group (CAc; **C,D**) and the NTc **(E,F)**. The nuclear organization and neuronal morphology of the 5-HT immunostained cells in the dorsal raphe complex is similar between groups. In images **(A,C,E)** dorsal is to the top with the midline in the middle of the image, while in images **(B,D,F)** dorsal is to the top and medial to the left. Scale bar in **(E)** = 250 μm and applies to **(A,C,E)**, scale bar in **(F)** = 100 μm and applies to **(B,D,F)**. DRif, dorsal raphe interfascicular nucleus; DRv, dorsal raphe ventral nucleus; DRd, dorsal raphe dorsal nucleus; DRl, dorsal raphe lateral nucleus; DRp, dorsal raphe peripheral nucleus.

For all mice in all three experimental groups, there were no marked differences in the location and morphology of the 5-HT+ neurons within the dorsal raphe nuclear complex (Figure 5). Within the dorsal raphe nuclear complex there were six distinct nuclei: the dorsal raphe interfascicular (DRif) nucleus, dorsal raphe ventral (DRv) nucleus, dorsal raphe dorsal (DRd) nucleus, dorsal raphe lateral (DRl) nucleus, dorsal raphe peripheral (DRp) nucleus and the dorsal raphe caudal (DRc) nucleus (Figure 5). The distribution of the serotonergic neurons forming these nuclei and their relationship to architectonic borders is similar to that previously described for laboratory rodents (e.g., Steinbusch, 1981), and thus a full description is not provided herein. The neurons of the DRp, DRl and DRc nuclei were readily distinguishable from the DRif, DRv and DRd nuclei since they were qualitatively substantially larger and multipolar (Figures 5B,D,F). Thus, for the neurons of the dorsal raphe complex, no differences in nuclear organization or neuronal morphology was detected between the three experimental groups investigated.

### Orexinergic Neurons of the Hypothalamus

**Figure 6:**
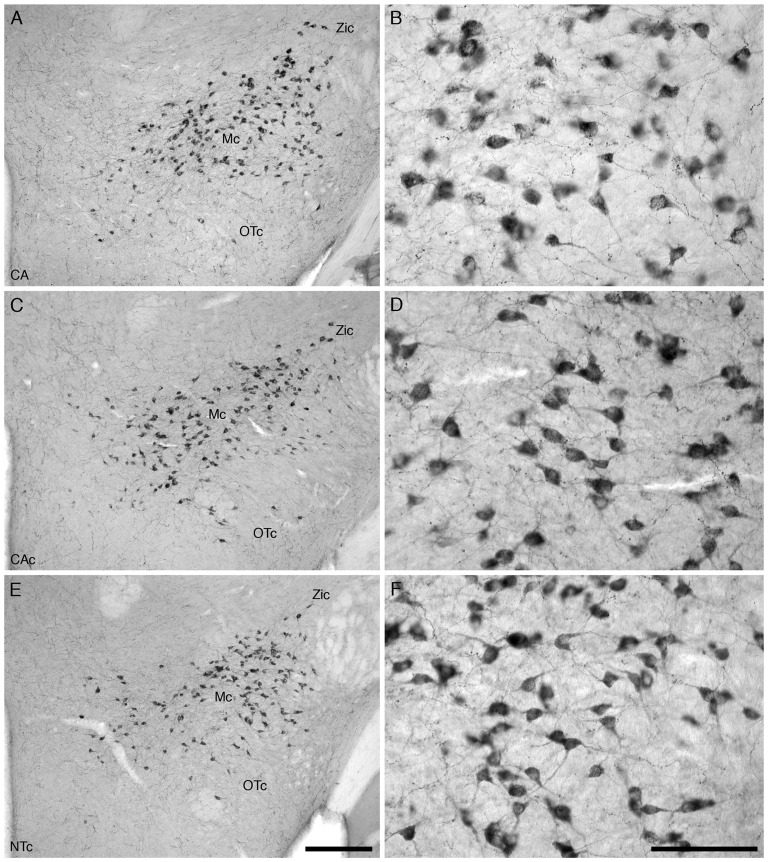
Lower **(A,C,E)** and higher **(B,D,F)** magnification photomicrographs of the hypothalamic orexinergic neurons of the mouse in the coronal plane immunostained for OxA in the three different groups analyzed in the present study, the group exposed to chronic prenatal alcohol (CA) **(A,B)**, the prenatal gavage control group (CAc) **(C,D)** and the NTc **(E,F)**. The nuclear organization and number of OxA immunostained cells was similar between groups, but the soma of the neurons in the CA group, exposed to chronic prenatal alcohol, were statistically significantly larger than the two control groups (CAc and NTc). In all images dorsal is to the top and medial to the left. Scale bar in **(E)** = 250 μm and applies to **(A,C,E)**, scale bar in **(F)** = 100 μm and applies to **(B,D,F)**. Mc, main orexinergic cluster; OTc, optic tract cluster; Zic, zona incerta cluster.

For all mice in all three experimental groups, there were no marked differences in the location and morphology of the OxA+ neurons within the hypothalamus (Figure 6). Within this aggregation of OxA+ hypothalamic neurons we could identify three clusters—a main cluster (Mc), a zona incerta cluster (Zic) and an optic tract cluster (Otc; Figure 6). The distributions of the neurons forming these clusters are similar to that reported in several rodent species previously and are thus not described in detail herein. All the OxA+ neurons were multipolar and exhibited a variety of somal shapes.

Our quantitative estimation of the numbers of OxA+ neurons in the hypothalamus of the three different experimental groups revealed a distinct homogeneity in numbers between individuals in the same group, and between groups. For the CA group, the average number of OxA+ neurons was 840.1 (s.e. 115.6) (male: 669.9 (s.e. 71.1), female: 1095.6 (s.e. 98.7)), for the CAc group it was 762.6 (s.e. 107.4) (male: 773.8 (s.e. 239.0), female: 751.5 (s.e. 21.7)) and for the NTc group it was 684.8 (s.e. 131.4) (male: 631.7 (s.e. 178.5), female: 738.0 (s.e. 227.1); Figure 2C). The number of OxA+ neurons was highest in the CA group, but statistical analyses using ANOVA and *post hoc* pairwise comparisons revealed that the number of OxA+ neurons in the CA group was not statistically significantly different from the CAc group (*p* = 0.892) or the NTc group (*p* = 0.639). A comparison between CAc and NTc groups was also not statistically significantly different (*p* = 0.891).

In terms of average somal area, the OxA+ neurons in the CA group had an average somal area of 63.3 μm^2^ (s.e. 1.3) (male: 66.7 (s.e. 2.0), female: 60.3 (s.e. 1.5)), for the CAc group it was 58.9 μm^2^ (s.e. 1.3) (male: 62.2 (s.e. 2.1), female: 56.2 (s.e. 1.6)) and for the NTc group it was 55.8 μm^2^ (s.e. 1.1) (male: 53.5 (s.e. 1.8), female: 57.5 (s.e. 1.4); Figure 3E). Statistical analyses using ANOVA and *post hoc* pairwise comparisons revealed that the OxA+ somal areas in the CA group were statistically significantly larger than the OxA+ neurons of the CAc group (*p* = 0.026) and the NTc group (*p* = 5 × 10^−5^). A comparison of somal area between the CAc and NTc groups was not statistically significantly different (*p* = 0.165; Figure 3E). In regards to average somal volume, the OxA+ neurons in the CA group exhibited an average somal volume of 426.3 μm^3^ (s.e. 13.1) (male: 457.0 (s.e. 20.9), female: 391.0 (s.e. 14.2)), for the CAc group it was 379.3 μm^3^ (s.e. 12.5) (male: 411.7 (s.e. 21.1), female: 352.9 (s.e. 14.6)) and for the NTc group it was 350.9 μm^3^ (s.e. 10.9) (male: 334.9 (s.e. 18.6), female: 363.2 (s.e. 13.0); Figure 3F). Similar to the somal area, statistical analyses revealed that the somal volume in the OxA+ neurons in the CA group was statistically significantly larger than the OxA+ neurons of the CAc group (*p* = 0.030) and the NTc group (*p* = 9 × 10^−5^). A comparison of somal volume between CAc and NTc groups was not statistically significantly different (*p* = 0.217; Figure 3F). Thus, for the hypothalamic orexinergic neurons, the only substantive difference observed between the groups was the increase in size, by around 7%–20%, of the soma in the group exposed to prenatal alcohol.

When comparing males between experimental groups, the somal areas of the NTc group were significantly different from the CA group (*p* = 3 × 10^−5^) and the CAc group (*p* = 6 × 10^−3^). In addition, the somal volumes of the NTc group were significantly different from the CA (*p* = 9 × 10^−5^) and the CAc (*p* = 0.021) groups.

### Orexinergic Boutons of the Cerebral Cortex

**Figure 7:**
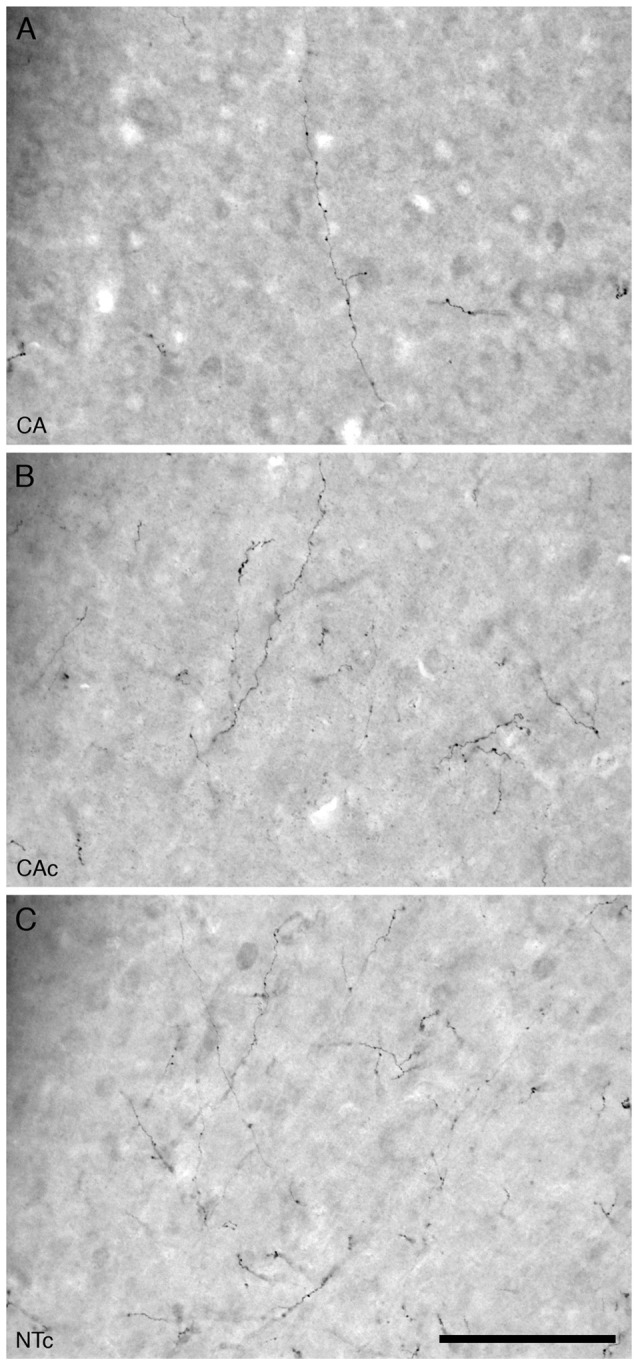
High magnification photomicrographs of the orexinergic axons and boutons in the anterior cingulate cortex of the mouse in the three different groups analyzed in the present study, the group exposed to chronic prenatal alcohol (CA; **A**), the prenatal gavage control group (CAc; **B**) and the NTc **(C)**. Note the lower density of orexinergic axons and boutons in the CA group compared to the two control groups (CAc and NTc). Scale bar in **(C)** = 100 μm and applies to all. In all images the pial surface is towards the top of the image.

For all mice in all three experimental groups, there were no marked differences in the location and morphology of the OxA+ boutons along the orexinergic axonal fibers in the anterior cingulate cortex (Figure 7). The ramifications of terminal axonal fibers were broadly distributed across all the cortical cell layers in the cerebral cortex and they appeared to have no specific spatial organization, although many traversed the cortex in a vertical manner. The OxA+ *en passant* boutons were distinct along the terminal axonal fibers and exhibited readily distinguishable small and large boutons as reported previously for this system (Dell et al., 2015). Qualitatively, in all three experimental groups, there appeared to be more small boutons than large boutons (Figure 7).

Our quantitative analysis of the density of OxA+ boutons in the anterior cingulate cortex of the three different experimental groups revealed a distinct homogeneity in numbers between individuals in the same group. For the CA group, the average density of OxA+ boutons was 60 boutons/mm^3^ (s.e. 4), for the CAc group it was 80 boutons/mm^3^ (s.e. 6) and for the NTc group it was 100 boutons/mm^3^ (s.e. 8; Figure 2D). The density of OxA+ boutons was lowest in the CA group, but highest in the NTc group. Statistical analyses using ANOVA and *post hoc* pairwise comparisons revealed that the number of OxA+ boutons in the CA group was statistically significantly different from the NTc group (*p* = 0.002) but not the CAc group (*p* = 0.081). A comparison between CAc and NTc groups was also not statistically significantly different (*p* = 0.136). Thus, it appears that the prenatal exposure to alcohol lowers the density of orexinergic boutons in the cerebral cortex.

## Discussion

In the present study we examined the nuclear organization, neuronal morphology, stereologically estimated total cell number and average cell size of four specific clusters of nuclei known to be involved in the control and regulation of the sleep-wake cycle. In addition, we examined the axonal bouton density of the orexinergic system in the anterior cingulate cortex. As well as differences between groups, we examined differences between the sexes within and between experimental groups; however, the differences between the sexes within and between groups were not systematic and are not discussed further. As outlined earlier, sleep in children suffering from fetal alcohol spectrum disorder (FASD) is perturbed in many ways, therefore a targeted analysis of some of the critical neuronal regulators of sleep was undertaken in mice exposed to a chronic prenatal alcohol regime to determine whether any clues to the basis for sleep disorders in FASD children could be found. For the most part, we did not record any major differences between mice exposed to prenatal alcohol and those not. The organization of the nuclei that form the pontine cholinergic, pontine catecholaminergic, midbrain serotonergic and hypothalamic orexinergic was similar across all mice studied. Furthermore, the specific neuronal morphology within the various nuclei was also unaltered by prenatal exposure to alcohol. The somal volumes and areas of the pontine catecholaminergic neurons of the LC were not affected by prenatal alcohol exposure; however, the somal volumes and areas of the pontine cholinergic and hypothalamic orexinergic did show significant variation in the group exposed to prenatal alcohol compared to both control groups. In addition, the density of orexinergic boutons in the anterior cingulate cortex was lower in the group exposed to prenatal alcohol than the control groups. These three specific differences are discussed in terms of the effect they may have on the control and regulation of the sleep wake cycle and how this relates to the observed sleep disorders in children with FASD.

### The Soma of the Pontine Cholinergic Neurons Are Smaller in Mice Exposed to Prenatal Alcohol

The first specific difference observed in the mice that underwent a chronic prenatal alcohol regime compared to the control mice was the smaller size (around 95%) of the pontine cholinergic neuronal soma of the LDT and PPT nuclei. The cholinergic neurons of the LDT and PPT are known to be involved in the generation of REM sleep, and the phasic events of REM sleep such as ponto-geniculo-occipital (PGO) spikes, through their projections to the thalamus and the basal forebrain (Webster and Jones, 1988; Semba et al., 1990). Additionally, these neurons are involved in the activation of thalamocortical systems by blocking synchronized oscillations such as seen in SWS (Paré et al., 1988). In the FASD mouse model generated in this study, these cholinergic neurons were reduced in size, which would hypothetically indicate that in the FASD model mice they would have a higher threshold potential, thus being less excitable, and being smaller, they may only be able to support a smaller axon and axon terminal field (Shepherd, 1979), making their action upon the recipient neurons less intense. Indeed, in experimental models where these neurons have undergone chemical ablation it was found that the amount of time spent in wake was significantly increased (Webster and Jones, 1988). Thus, it appears that the difficulty experienced in going to sleep, short sleep durations and increased sleep disruptions observed in FASD children (Meltzer and Mindell, 2004; Jan et al., 2010; Wengel et al., 2011) may in part be related to a reduction in size of the cholinergic neurons of the LDT and PPT.

### The Soma of the Hypothalamic Orexinergic Neurons Are Larger in Mice Exposed to Prenatal Alcohol, but Bouton Density in the Anterior Cingulate Cortex Is Lower

The second specific difference observed in the mice treated with a chronic prenatal alcohol regime when compared to the control groups was the significantly larger size of the soma of the hypothalamic orexinergic neurons. Interestingly, the third specific difference observed was that the orexin bouton density in the cerebral cortex was lower in the mice treated with a chronic prenatal alcohol regime. The hypothalamic orexinergic neurons are known to project throughout the entire central nervous system, but exhibit specifically intense projections to other regions of the brain involved in arousal (e.g., Peyron et al., 1998). Thus, orexin (or hypocretin) has been implicated in arousal as one of the main functions of this system (e.g., Saper et al., 2001; Siegel, 2004a,b). Many of the disorders associated with sleep in FASD children appear to be related to problems associated with arousal, and thus the arousal systems of the brain (Meltzer and Mindell, 2004; Haydon et al., 2009; Jan et al., 2010; Wengel et al., 2011). In this sense, the changes observed in the orexinergic system in the currently used mouse model of FASD is of interest—perhaps the changes observed in this mouse model can help explain some of the sleep disturbances observed in FASD children.

The orexinergic neurons in the FASD mouse model were approximately 1.1–1.2 times larger than those measured in the control mice. One of the key cellular level phenomena associated with an increase in neuron size, and thus an increase in neuronal surface area, is a lower threshold potential (Shepherd, 1979). Thus, the larger orexinergic neurons found in the FASD mouse model would, theoretically, be more readily excitable when compared to the control groups. In this sense, the brain globally, and in particular the arousal systems receiving intense projections from the orexinergic neurons of the hypothalamus, would potentially be in receipt of a greater number of excitatory action potentials from the hypothalamus in the FASD mouse model than the controls. This may augment the arousal function of the orexinergic neurons. The second key feature associated with increased neuron size would be the theoretical ability of the larger cell to support a larger and more extensively branched axon and axonal bouton terminal field (Shepherd, 1979), but this was not observed. Indeed, the contrary was observed, at least in the anterior cingulate cortex, where the orexin bouton density was lower in the group exposed to a chronic prenatal alcohol regime than the controls. Interestingly, in previous studies of orexinergic cortical projections in Cetartidoactyls, the cetaceans had a lower density of orexinergic boutons in the cerebral cortex despite having greater numbers of smaller neurons than artiodactyls (Dell et al., 2012, 2015). The cetaceans are under pressure to maintain arousal in their aquatic environment and through the manner in which they sleep. Thus, while the differences in the orexinergic system of the mice exposed to chronic prenatal alcohol cannot directly explain the disorders of arousal observed in FASD children, the difference observed do indicate that there may be problems with the arousal system in FASD children.

### Further Studies

In the current study we have observed three specific differences in two distinct neuronal systems involved in the regulation and control of the sleep-wake cycle. These three differences, when viewed in light of previous experimental studies of the sleep-wake cycle, appear to be related to the disorders of sleep experienced in children suffering from FASD. It would appear that prenatal alcohol exposure causes deficits within the arousal systems of both the hypothalamus (orexinergic) and pons (cholinergic), and that these deficits may cause the disorders observed in FASD children. It would be useful in future to undertake polysomnographic recording of sleep in the FASD mouse model and FASD children to determine to what degree the sleep disorders in the FASD children match any potential sleep disorders in the FASD mouse model, as well as examine in detail potential changes to the terminal projection fields of these systems in various regions of the mouse brain. Given the findings of the current study and the information to date for sleep in FASD children, REM sleep would appear to be an interesting target for study. Given the success in treatment of sleep disorders, such as narcolepsy (Didato and Nobili, 2009), further research into the precise mechanism causing sleep disorders in FASD models and children may open avenues for the use of neurotherapeutics to ameliorate and potentially alleviate sleep disorders associated with FASD. In future, such symptoms as night terrors (Durmer and Dinges, 2005), sleep walking (Randazzo et al., 1998) and daytime tiredness (Lancioni et al., 1999) may be treatable in FASD children and thus increase their quality of life significantly by keeping these symptoms under control.

## Author Contributions

OIO, AOI and PRM conceptualized the study. OIO, AB and PRM undertook the practical work and analysis. OIO and PRM wrote the first draft of the article and subsequently edited it with the suggestions of AB and AOI.

## Conflict of Interest Statement

The authors declare that the research was conducted in the absence of any commercial or financial relationships that could be construed as a potential conflict of interest.
